# Foodborne *Campylobacter*: Infections, Metabolism, Pathogenesis and Reservoirs

**DOI:** 10.3390/ijerph10126292

**Published:** 2013-11-26

**Authors:** Sharon V. R. Epps, Roger B. Harvey, Michael E. Hume, Timothy D. Phillips, Robin C. Anderson, David J. Nisbet

**Affiliations:** 1 Food & Feed Safety Research Unit, Southern Plains Agricultural Research Center, Agricultural Research Service, United States Department of Agriculture, 2881 F&B Road, College Station, TX 77845, USA; E-Mails: roger.harvey@ars.usda.gobv (R.B.H.); michael.hume@ars.usda.gov (M.E.H.); david.nisbet@ars.usda.gov (D.J.N); 2 Veterinary Integrative Biosciences, College of Veterinary Medicine, Texas A&M University, College Station, TX 77845, USA; E-Mails: drsepps@tamu.edu (S.V.R.E.); tphillips@cvm.tamu.edu (T.D.P.)

**Keywords:** *Campylobacter coli*, *Campylobacter jejuni*, diarrhea, foodborne pathogen, Guillain Barré syndrome

## Abstract

*Campylobacter* species are a leading cause of bacterial-derived foodborne illnesses worldwide. The emergence of this bacterial group as a significant causative agent of human disease and their propensity to carry antibiotic resistance elements that allows them to resist antibacterial therapy make them a serious public health threat. *Campylobacter jejuni* and *Campylobacter coli* are considered to be the most important enteropathogens of this genus and their ability to colonize and survive in a wide variety of animal species and habitats make them extremely difficult to control. This article reviews the historical and emerging importance of this bacterial group and addresses aspects of the human infections they cause, their metabolism and pathogenesis, and their natural reservoirs in order to address the need for appropriate food safety regulations and interventions.

## 1. The Genus *Campylobacter*

Bacteria belonging to the genus *Campylobacter* are Gram-negative, spiral, non-spore forming rods that form spherical or coccoid bodies in older cultures [1]. They are between 0.2 to 0.9 microns wide and 0.5 to 5 microns long, are motile and usually move with a polar unsheathed flagellum at one or both ends, and are microaerobic with a respiratory-type metabolism, although there are some that grow aerobically or anaerobically [2]. The sequence of the genome of *Campylobacter jejuni* NCTC11168 was originally published in 2000 [3]. There is considerable variation between strains and re-annotation of the *C. jejuni* genome published in 2006 revealed that the complete sequence is 1,641,481 bp in length with 25 polymorphic regions [4]. Moreover, new information for 1,450 of the original 1,654 coding sequences revealed changes corresponding to over 300 product functions [4]. The infectious diseases caused by members of the bacterial genus *Campylobacter* are called campylobacteriosis [5]. Currently *Campylobacter jejuni* and *Campylobacter coli* are considered to be the most important enteropathogens among *Campylobacter* spp. [6]. The rate of *Campylobacter* infections are increasing worldwide, exceeding shigellosis [5,7].

## 2. *Campylobacter* Historical Perspective

From a historical standpoint, the first report of *Campylobacter* is believed to have been made in 1886 by Theodore Escherich, who observed and described a non-culturable spiral shaped bacteria, which he found in the colon of children with an enteric disease called “cholera infantum” [8,9,10,11]. *Campylobacter* was identified February 2, 1906 by two British veterinarians, John McFadyean and Stewart Stockman who reported the presence of “large numbers of a peculiar organisms” in Loeffler’s blue-stained smears of uterine mucus from a pregnant ewe [12]. In 1927, a group of vibrio-like bacteria was found in the feces of cattle with diarrhea. They were described by Theobold Smith and Marion Orcutt [6,13]. In 1931, Jones and coworkers showed a relationship between the microaerophillic vibrios and bovine dysentery, and the organism was eventually called *Vibrio jejuni*. The first well documented incident of *Campylobacter* infection took place in Illinois in 1938. The case involved a milk-borne outbreak of diarrhea that affected 355 inmates in two adjacent state institutions [14,15]. In 1944, Doyle isolated another vibrio from the feces of pigs with diarrhea and classified it as *Vibrio coli* [16,17]. Because of their low DNA base (low guanine and cytosine) composition, non-fermentative metabolism and their microaerophillic growth requirements the genus *Campylobacter* was proposed by Seabald and Vernon in 1963, distinguishing them from the *Vibrio* spp. [9,18]. There are two subspecies recognized within *C. jejuni*, *C. jejuni* subspecies *jejuni* and *C. jejuni* subspecies *doylei*. Strains of *C. doylei* differ from *C. jejuni* biochemically in its inability to reduce nitrate and variable growth at 42 °C [19]. *Campylobacter coli* and *C. jejuni* differ biochemically in their ability to hydrolyze hippurate. *Campylobacter coli* cannot hydrolyze hippurate and there are some *C. jejuni* subspecies that are hippurate negative [6].

## 3. Human Infections

*Campylobacter* are a leading bacterial cause of zoonotic disease worldwide, affecting approximately 1% of the human population in Europe each year [20] and infecting 13 of every 100,000 persons in the United States annually [21]. Clinical manifestations of campylobacteriosis are largely undistinguishable from other bacterial gut infections and include fever, abdominal cramping, and diarrhea, with or without blood in stools [6,22]. These symptoms can last from three days to one week [23]. In severe cases of *C. jejuni* infection, individuals may exhibit fever, abdominal cramps and diarrhea that contain blood and leukocytes, or they may develop post infection complications associated with Guillain Barré Syndrome or Miller Fischer Syndrome. Miller Fischer Syndrome is a subform of Guillain Barré Syndome characterized by areflexia, ataxia and opthalmoplegia [6,24]. *Campylobacter* can also cause post-infection complications associated with acquired immune-mediated neuropathies such as Guillian Barré Syndrome or Miller Fischer Syndrome [25,26]. In the developed world, most human *Campylobacter* infections are caused by *Campylobacter jejuni*; however, 4% of clinically confirmed cases in one study were attributed to *Campylobacter coli* [27]. Campylobacteriosis can occur in all age groups and the cost of human illness in the United States is estimated at $1.3 to 6.8 billion dollars annually [27,28].

In March of 2013 the Centers for Disease Control and Prevention (CDC) reported that in 2012 there was a 14% increase in the incidence of food-borne illness caused by *Campylobacter jejuni* [29]. In 2005, the CDC estimated that the number of campylobacteriosis cases in the United States was about 1 million per year making it the second leading cause of laboratory confirmed cases of foodborne illness in the United States thus resulting in an estimated 12.72 cases per 100,000 people [30]. In 2009, the Foodborne Disease Active Surveillance Network (FoodNet) of the CDC estimated that the number of infections by *Campylobacter* was a total of 6,033, or 13.02 per 100,000 people [9]. In the last five years the European Food Safety Authority (EFSA) and the European Centre for Disease Prevention and Control (ECDC) reported that campylobacteriosis has become the most often reported zoonosis in the European Union followed by salmonellosis and yersinosis [9,31,32]. More than 200,000 confirmed cases of campylobacteriosis were reported in 24 of the member states of the European Union at a rate of 45.2 cases per 100,000 people. In 2010 New Zealand reported the highest national campylobacteriosis rate, which peaked in May 2006 at 400 per 100,000 population [9]. In general, developing countries do not have national surveillance programs for campylobacteriosis; consequently, there are no case incidence values in terms of population density [7]. Most of the estimates of incidence are done by laboratories where the surveillance is based on pathogens responsible for diarrhea*.*

*Campylobacter* isolation rates in developing countries range from 5 to 20% [7,33]. Most of the data was collected by the World Health Organization (WHO), which along with the Canadian Public Health Service have provided financial support to developing countries for epidemiologic studies [7,34]. There is a large disparity in the incidence of campylobacteriosis in developing countries *versus* that of developed countries. High numbers of children in developing countries are affected by *Campylobacter* infections. Community based studies in developing countries have estimated that 60,000 per 100,000 children <5 years of age [7,34,35]. This data suggests that campylobacteriosis is a pediatric disease in developing countries. In developed countries, more than 90% of human campylobacteriosis cases occur during the summer because of undercooked meats from outdoor cooking facilities. People of all ages are affected, but particularly children less than 4 years of age and young adults 15–44 years of age [14,28]. 

The infectious dose of *Campylobacter jejuni* for humans is estimated to be low; between 500–800 organisms [6]. The dosage was estimated in 1981 as a result of a human experiment in which a British medical doctor, Robinson, swallowed 500 organisms of known serotype in 180 mL of pasteurized milk [6,36]. The findings from this self-inflicted experiment, which reportedly satisfied the criteria of Koch’s Postulates and the findings of additional human experiments, verified a dosage and a mechanism of *C. jejuni* human infection [6].

*Campylobacter* are also recognized as reservoirs for antimicrobial resistance genes that potentially can be exchanged between other pathogenic and commensal bacteria [37,38,39,40]. A 2011 review article hypothesizes that decades of indiscriminant use of antibiotics in food animal production may have led to the emergence and spread of antibiotic resistance among *Campylobacter* spp. [41]. The evidence of this is strongly supported [9]. 

Antibiotics have been used for decades in food production animals to control, prevent, and treat infections and to enhance growth [9,42,43]. This usage has caused an increased resistance to multiple antibiotics by members of *Campylobacter* spp. in food production animals and environments [9,44]. The development of antimicrobial resistance in *Campylobacter* spp. is a serious threat to human health [9]. Worldwide, there has been a rapid increase in the proportion of *Campylobacter* strains resistant to antimicrobial agents [37,45,46,47]. *Campylobacter jejuni* and *C. coli* are almost intrinsically resistant to penicillians, cephalosporins (with exception of a few 3rd generation cephalosporins), trimethoprim, sulfamethoxazole, rifampicin and vancomycin [6]. For patients infected with *Campylobacter* spp., the prognosis is that most will recover without specific treatments other than replacement of fluid and electrolytes, however, in severe cases antibiotics such as macrolides and fluroquinolones are generally administered [9]. It is believed that increasing resistance to fluroquinolones and erythromycin by *C. jejuni* and *C. coli* might compromise the effectiveness of these treatments [37,47,48,49].

## 4. *Campylobacter* Metabolism

Although there has been a significant amount of knowledge elucidated about the *Campylobacter* genus, there is still more to learn about *Campylobacter’s* metabolic processes. For instance, the process of nutrient acquisition by *Campylobacter* has not been completely elucidated but it is apparent this process is flexible enough to allow survival in the environment (water, food or feces) before host ingestion [50]. Unlike many other gut bacteria, however, *Campylobacter* are limited in their ability to conserve energy for growth and maintenance via fermentation of carbohydrates. These bacteria lack 6-phophofructokinase, which is a key enzyme in energy metabolism [51], although a novel L-fucose pathway has been elucidated by Muraoka and Zhang [52] and Stahl [53]. Moreover, *Campylobacter* are further limited in their ability to conserve energy for growth via fermentation of carbohydrates due to the absence an active phosphoenolpyruvate dependent phosphotransferase system, which would function to transport and phosphorylate sugars simultaneously [6,51]. *Campylobacter* can conserve energy via respiration, oxidizing hydrogen and formate for the reduction of the electron acceptors (fumarate, nitrate, sulfites) and, if at low concentrations, oxygen, to generate proton motive force for electron transport phosphorylation [51,54,55,56,57]. *Campylobacter jejuni* relies on the use of the amino acids and the citric acid cycle intermediates as carbon sources [58]. *Campylobacter jejuni* utilize available amino acids in a sequential order, with preference to, serine, aspartate, asparagine, and glutamate and other *C. jejuni* strains have been found to metabolize proline but only after all other amino acid sources have been completely utilized [50]. *Campylobacter* can conserve energy via respiration, oxidizing hydrogen and formate for the reduction of electron acceptors such as fumarate, nitrate, sulfites and if at low concentrations, oxygen, to generate proton motive force transport.

There are some *C. jejuni* subspecies *doylei* which contain some enzymes from the Entner-Doudoroff pathway which convert glucose-6-phosphate to glyceraldehyde-3-phosphate utilizing gluconate-6-phosphate instead of a fructose-6-phosphate intermediate. This pathway eliminates the need for phosphofructokinase [6]. It has generally been assumed that *C. jejuni* were found to contain genomic islands (cj0480c-cj0490) that are up regulated in the presence of L-fucose and mucin obtained from the host during colonization in the intestine [53]. The current knowledge of *C. jejuni in vivo* metabolism is based on amino acid utilization to support the growth and establishment of colonization in the host intestines. *Campylobacter jejuni*’s metabolic diversity is evidenced by its differential carbon utilization [52,59,60].

## 5. *Campylobacter* Pathogenesis and Disease

The exact sequential steps of *C. jejuni* infection to *C. jejuni* mediated enteritis are unknown. What is known are the requirements for *C. jejuni* virulence: (1) motility, (2) drug resistance, (3) host cell adherence, (4) host cell invasion, (5) alteration of the host cell signaling pathways, (6) induction of host cell death, (7) evasion of the host (8) immune system defenses, and (9) acquisition of iron which serves as a micronutrient for growth and works as a catalyst for hydroxyl radical formation [61,62,63,64,65,66]. It is also known that *C. jejuni* secretes proteins that contribute to the ability of the bacterium to invade the host epithelial cells [61]. Survival in the host environment depends on several adaptive responses including adherence, protein secretion, invasion and replication. 

The biochemical effects on cellular events are as follows: (1) cytoskeletal rearrangement, (2) host cell death, (3) tight junction disruption and cytokine induction that leads to loss of epithelial cell function, (4) a compromised barrier and absorptive functions, and (5) tissue destruction and disease manifestation. Adherence can also lead to an early inflammatory response that causes stimulation of innate immune functions in the following sequence: (1) an influx of fluid, (2) complement cell activation, (3) recruitment of phagocytes that can lead to *C. jejuni* lysis or death, (4) clearing of the infection or the presentation of antigens, (5) a humoral response and, (6) a clearing of the infection. In the other direction, instead of *C. jejuni* lysis or death, the inflammatory response could lead to tissue destruction and disease manifestation [61].

There is evidence that *C. jejuni* infections commonly precede Guillain Barré Syndrome which results from a case of molecular mimicry whereby host produced anti-*Campylobacter* antibodies recognize and cross react with self gangliosides and damage peripheral nerve tissue [6]. Guillain Barré Syndrome is a rare autoimmune disease characterized by the demyelination of motor and sensory nerves or deterioration of axonal nerves of the peripheral nervous system. This nerve damage can lead to muscle weakness, paralysis, and death. It is reported that Guillain Barré Syndrome “has become the most frequent cause of acute flaccid paralysis since the near elimination of poliomyelitis in the world” [26,67,68,69]. 

Studies have established Guillain Barré Syndrome as a mechanism of molecular mimicry based on Koch’s and Witebsky’s postulates. They are as follows: (1) the establishment of an epidemiological association between the infectious agent and the autoimmune disease, (2) the identification of T cells or antibodies directed against the patient’s target antigens, (3) the identification of microbial mimics of the target antigen, and (4) reproduction of the disease in an animal model. Autoantibodies are the pathogenic component that triggers Guillain Barré Syndrome [68]. 

There are two major sub-forms of Guillain Barré Syndrome that affect the peripheral nervous system. The major sub-forms of Guillain Barré Syndrome are: (1) acute inflammatory demyelinating polyneuropathy characterized by the demyelination of peripheral nerves, (2) acute motor axonal neuropathy characterized by degeneration of axonal components of peripheral nerves, and (3) Miller Fischer syndrome characterized by areflexia, ataxia, and ophthalmoplegia. Studies have indicated that *C. jejuni* infection precedes Guillain Barré Syndrome in 20 to 50% of cases in Europe, North and South America, Japan, and Austraila. The percentage is suspected to be higher in developing countries. Since *Campylobacter* infections occur far more frequently than Guillain Barré Syndrome, neither the characteristics of the host nor the strain of *Campylobacter* species are known determinants of which persons with *Campylobacter* infection contract Guillain Barré Syndrome. Guillain Barré Syndrome usually develops 1 to 3 weeks after a *C. jejuni* infection. Studies in Japan have indicated that the risk of developing Guillain Barré Syndrome may be higher after infection with *C. jejuni* type O:19 [6,26,67].

## 6. Reservoirs of *Campylobacter*

In developing countries *Campylobacter* infection is said to be hyperendemic and major sources of infection are environmental and food contamination [7]. Conversely, food production animals are considered to be the primary source of *Campylobacter* infections in humans in developed countries such as the United States. From animal farms to the commercial production of food commodities there are numerous possibilities for transmission of *Campylobacter* infection through cross-contamination. *Campylobacter* infection from consumption of poultry, beef, and pork products is the leading cause of human foodborne illness. Poultry is estimated to account for 50%–70% of human *Campylobacter* infection. Poultry includes broilers, laying hens, turkeys, ducks, and ostriches [6]. Studies have indicated that the prevalence of *Campylobacter* colonization in cattle is 0%–80% and 20% in sheep. The same study reported that pigs were more contaminated than cattle or sheep [70]. Poultry and poultry products are considered to be the largest contributor of human *Campylobacter* infection. *Campylobacter jejuni* mainly colonizes poultry and is found predominantly in the cecum and colon, but it can also be found in the crops. Horrocks *et al.* speculated that due to the higher metabolic temperatures of the poultry species they are possibly predisposed to become prominent reservoirs for the thermotolerant *C. jejuni* [71].

*Campylobacter* transmission is prevalent during preharvest conditions. Colonization in broiler chicks has been demonstrated to be a risk factor in horizontal transmission of *C. jejuni* infection. Broiler chick colonization is estimated to take place no sooner than seven days of age [70,72]. Other studies have found that broiler chick colonization takes from zero to three weeks. Colonization of free-ranging chickens is estimated to take place from 0–8 days [71,73,74,75]. Although young animals are very susceptible to colonization by *C. jejuni*, older broiler chicks closer to processing age have a higher percentage of *Campylobacter* colonization. Despite horizontal transmission of the flock taking place rapidly, colonization of the flock can take up to several weeks [71]. Studies have estimated that up to 98% in the United States and 60% to 80% in Europe of retail chicken meat is contaminated with *C. jejuni* [73]. It was found that the skin and giblets have particularly high concentrations of *Campylobacter* contamination [73].

Despite the consistent implication that *Campylobacter* colonization of poultry and poultry products is the major contributor to human foodborne illness, cattle and other swine frequently carry *C. jejuni* and *C. coli* [76,77,78,79,80,81]. It is probable that cattle carcasses are contaminated during processing either directly or indirectly. None the less, the cases of human foodborne illness as a result of *Campylobacter* contamination in bovine products (unpasteurized milk and meat) is a legitimate concern [76,77].

Multiple studies with cattle have shown that *Campylobacter* preferentially colonize the lower gastrointestinal tract as opposed to the upper gastrointestinal tract (the 1st stomach) where there is a lower pH environment in the rumen [78]. Additionally the stratified epithelium of the rumen probably lacks the necessary receptors to sustain a persistent high level of *Campylobacter* colonization [78]. The gallbladder, liver and bile have all been shown to harbor moderately high percentages of *Campylobacter*. In recent studies, 33% and 21.8% respectively of samples tested positive for *Campylobacter* in the gallbladder, its mucosal tissue and bile [71,79,80,81]. Unpasteurized bovine milk and milk products are common vehicles for *Campylobacter* foodborne disease transmission. In a study with cattle from a dairy farm 12% of raw milk samples were found to be contaminated with *C. jejuni* possibly as a result of contact with bovine feces, contaminated water or direct contamination as a result of mastitis [41].

The incidence of *Campylobacter* is reported to be lower for forage fed (primarily dairy cattle) than that of feedlot cattle. The possible contributors to the high incidence of *Campylobacter* in feedlot cattle are: (1) increased stocking densities, (2) constant contact with feces from other animals, and (3) the high frequency of shared access to community feeding and water troughs [71,82]. These studies found that there was a higher prevalence of *Campylobacter* in feedlot cattle (68%) compared to that of adult cattle from the pasture (7.3%) and others found that prevalence rates increased in fed cattle from 1.6% near or upon entry to the feedlot to 63% near the end of the finishing period [76]. Prevalence numbers remained relatively the same whether tests were conducted before or after transport to processing [82]. These results suggest that confinement may promote increased carriage and horizontal transmission of *Campylobacter*. Further swab tests on the hides of these cattle yielded the same results. The feedlot cattle had significantly more contaminated hides [82].

The numbers of swine that are colonized by *Campylobacter* are more comparable to cattle than to those of poultry. *Campylobacter coli*, which normally inhabit pig intestines, are also found on pork products. Swine are predominantly colonized with *C. coli* albeit less frequently they are colonized with *C. jejuni* [79]. Young *et al.* [80] demonstrates that despite the prevalence of *C. coli* in swine, a high prevalence of *C. jejuni* enteric colonization (cecal or rectal contents) has been observed in gilts, sows, and weaned piglets. Studies conducted by Jensen *et al.* [71,81] investigated *C. jejuni versus* *C. coli* colonization of outdoor organically-reared pigs to monitor possible shifts from *C. coli* to *C. jejuni* intestinal colonization. Their findings from three research trials indicated that there were excessive fluctuations (0%, 18.8% and 78.6%) of swine colonized by *C. jejuni*, and in each trial swine were still dominantly colonized by *C. coli* [81]. There have also been research studies that demonstrate the possible co-existence of *C. coli* and *C. jejuni* in pigs with *C. jejuni* being present in lower numbers [81,83]. In these studies; however, *C. jejuni* was never found to be more prevalent than *C. coli*.

The life of swine raised for food production starts at a farrowing barn where they are born. After three weeks they are moved to a nursery where they will stay until they reach 22.6 kg (6 weeks). Next they are moved to a finishing unit where they will stay until they reach market growth rate (99.8–113.4 kg). Environmental conditions naturally play an important role in *Campylobacter* transmission in swine the same as in cattle and poultry. There are increased possibilities for *Campylobacter* infection and disease transmission in finishing units and breeding farms [80]. There are distinct differences in the prevalence and occurrence of *Campylobacter* colonization and infection of pigs raised in organic outdoor production systems than those raised in conventional farm systems [84].

## 7. Conclusions

Recent reported increases of *Campylobacter* infections attributed to foodborne illness in the United States and elsewhere indicate that *Campylobacter* continues to be an emerging pathogen worldwide. Therefore it is imperative that the food industries recognize the increased persistence of *Campylobacter* infections as a serious public health risk and take measures to improve food safety by making pre and post-harvest practices more stringent. Efforts to reduce or eliminate *Campylobacter* contamination in the food industry needs to become a more determined priority. Poultry and poultry products remain the number one cause of foodborne illness worldwide. Effective pre-harvest interventions that reduce the concentration and prevalence of *Campylobacter* in food producing animals are needed for all animal species. Current post harvest interventions are limited to non-specific hygienic controls and new and more specific interventions are needed to help reduce product contamination. The consumption of undercooked poultry products and the cross-contamination of carcasses and other food products continues to be a major public health concern. The continual emergence of antibiotic resistant *Campylobacter* infections poses a serious public health concern in addition to the fact that there are still many aspects about the *Campylobacter* species physiology that we have not elucidated. All of the factors stated above increase the need for more stringent food safety regulations, new research and application of new and improved pre-harvest and post-harvest strategies.
